# Challenges in higher education: Differences between students with and without specific learning disorder and the moderating role of executive functions

**DOI:** 10.1371/journal.pone.0331375

**Published:** 2025-09-05

**Authors:** Marlyn Khouri, Atheer Massarwe, Noga Cohen

**Affiliations:** 1 Department of Special Education, Faculty of Education, University of Haifa, Haifa, Israel; 2 The Edmond J. Safra Brain Research Center for the Study of Learning Disabilities, University of Haifa, Haifa, Israel; Anurag University, INDIA

## Abstract

Students with Specific Learning Disorders (SLD) face difficulties not only in academic skills but also in the social, emotional, and executive function (EF) domains. These challenges may increase vulnerability to rumination—a repetitive and maladaptive focus on distress, which is strongly linked to emotional difficulties. This study explores differences in academic, social, emotional, and EF challenges between students with and without SLD and investigates whether these challenges moderate the relationship between SLD and rumination. A sample of 95 college students (46 with SLD, 49 without SLD) completed questionnaires assessing rumination and emotional distress. They also completed a writing task in which they provided an upsetting event related to their studies. The event were coded to four categories related to students’ challenges: academic, social, emotional, and EF challenges. Results indicated that students with SLD reported significantly higher levels of rumination, greater EF challenges, and higher levels of perceived stress than their peers. A moderation analysis further revealed that EF difficulties significantly moderated the link between SLD and rumination, with students experiencing both SLD and EF challenges showing the highest rumination levels. Findings emphasize the critical role of EF in the emotional experiences of students with SLD. Addressing EF deficits among students with SLD can reduce emotional distress and improve academic outcomes.

Specific Learning Disorders (SLD) are neurodevelopmental conditions that affect academic skills, such as reading, writing, and math, despite normal intelligence and education [1,2]. Recently, the number of students with SLD entering postsecondary institutions is mounting [3–6]. These students face significant challenges that go beyond academic difficulties [e.g., 7–9], affecting their social, emotional, and psychological well-being [10–13]. Additionally, they struggle with cognitive difficulties and executive function (EF) challenges [e.g., 9,14–16]. These challenges can profoundly impact their overall well-being [8–9], as well as their self-efficacy [17,18] and academic performance [17].

Emotion regulation (ER) plays a key role in managing these challenges. Students with SLD often rely on maladaptive ER strategies, such as rumination [e.g., 19], a repetitive thinking about one’s problems and negative emotional experiences [20]. Rumination negatively affects students’ well-being and self-efficacy [17,18,21–23], potentially leading to depression and academic struggles [24].

The Vulnerability-Stress Model [25] offers a framework for understanding how a range of vulnerabilities associated with SLD—including cognitive, emotional, and social factors—interact with environmental stressors to heighten emotional difficulties, with rumination exacerbating distress and impeding adaptive coping. This study therefore examined four major challenges faced by SLD students—academic, social, emotional, and executive function (EF)—and their potential link to rumination. While it is known that students with SLD often encounter various difficulties in higher education, there is a lack of information regarding how these distinct challenges are associated with maladaptive emotional processes such as rumination. Therefore, the main goal of the current study is to explore the relationship between these challenges and rumination.

In terms of academic difficulties, students with SLD often face academic settings that require high-level literacy, analytical thinking, and self-regulatory abilities—domains frequently impacted by their learning difficulties [8]. In particular, they may experience persistent challenges in reading comprehension, such as difficulties with decoding, fluency, and processing complex material, which can result in slower reading speeds and greater cognitive strain [7–9]. Writing is another area of struggle, as issues with spelling, grammar, and the coherent expression of ideas can interfere with producing well-organized texts and effectively recording information during lectures [26–28]. Furthermore, students with SLD are often not equipped with the necessary academic strategies to manage the intensive demands of higher education [7,27]. The rigorous expectations of college-level coursework and the complexity of assignments tend to intensify these academic challenges [7,8]. These challenges may be associated with maladaptive coping patterns, such as being stuck on a ruminative cycle [29]. However, this was not tested among college students with SLD.

In addition to academic pressures, students with SLD often encounter significant social difficulties. They may find it challenging to interpret social cues, initiate friendships, and sustain interpersonal connections, which can contribute to feelings of loneliness and social isolation [12,13]. Moreover, individuals with SLD are more susceptible to experiences of bullying and peer rejection, negatively impacting their self-esteem and emotional health [30–32]. These challenges foster feelings of inadequacy [32], increasing vulnerability to stress, and rumination on interpersonal interactions [19,33]. However, the idea that social difficulties among students may be related to rumination was only tested among children and not among college students with SLD.

In terms of emotional challenges, students with SLD experience higher levels of stress, anxiety, and depression compared to their non-SLD peers [10,11,34]. Moreover, they demonstrate low self-esteem [35, 36], as they feel inferior to their peers, what may exacerbate emotional distress and negatively affects their academic success [34]. ER challenges are also prevalent among SLD students, making it difficult for them to manage negative emotions and stress in academic and social contexts [10]. This emotional dysregulation can exacerbate feelings of frustration and helplessness, particularly when faced with academic challenges, contributing to a cycle of negative emotions that hinders their ability to engage effectively in learning [8]. This sensitivity to stressors like academic failure and social rejection may trigger ruminative thought patterns [10,36,37]. To the best of our knowledge, the direct relationship between these challenges and rumination has not yet been explored among students with SLD.

Students with SLD also demonstrate EF difficulties, including organization, planning and time management, which are key to academic success [9,16,18,38]. These challenges can manifest in their inability to efficiently manage time, set academic goals, or complete assignments [9,16]. Research highlights that students with SLD often struggle with working memory, cognitive flexibility, and inhibitory control, which are key components of EF [14,15]. For instance, they may find it challenging to shift their attention from one task to another or to suppress irrelevant information, leading to difficulties in adapting to new academic demands [39]. Such deficits make it harder for students with SLD to keep pace with academic requirements, organize their workload, or manage multiple assignments simultaneously, often resulting in increased frustration and stress [40]. EF deficits hinder coping strategies, the ability to disengage from negative thoughts [20,41–44], and contribute to rumination [41,42], affecting both academic performance and well-being [45–47]. While prior research showed a link between EF and rumination among healthy adults and clinical populations [41,42], this link was not studied in the context of SLD.

This aim of the current study was to compare academic, social, emotional, and executive function (EF) challenges, as well as rumination levels and emotional distress (psychological distress and perceived stress) among students with and without SLD. Furthermore, the study explored whether these challenges moderate the link between SLD and rumination. Based on previous research showing heightened academic, social, emotional, and cognitive challenges among students with SLD [4,9], we predicted that students with SLD will report significantly more challenges in these domains than students without SLD. Based on prior findings [19,41,42], we also predicted that students with SLD will show higher rumination and emotional distress levels. Finally, past research showed a strong link between stress and rumination, with academic challenges intensifying this connection by increasing focus on failures and perceived inadequacies [48,49]. Based on these findings, we predicted that academic, emotional, social, and EF challenges would moderate the relationship between SLD status and rumination—such that students with SLD who experience greater difficulties in these areas would report higher levels of rumination compared to those with fewer challenges.

## Materials and methods

### Participants

A total of 104 college students from various Israeli universities and colleges participated in this study, including students with and without SLD. Nine participants were excluded due to task issues, leaving a final sample of 95 students (46 with SLD, 49 without SLD), with an average age of 25.05 years (SD = 2.82). Most participants were women (73.7%) undergraduates (86%). The study followed APA ethical guidelines and was approved by the University of Haifa’s ethics committee (approval number 068/20).

A post hoc power analysis using G*Power [50] for independent t-tests indicated that, with a total sample size of 95 participants, the study had 67% power. The analysis was conducted with an alpha level of *p* = .05 and a medium effect size (Cohen’s *d* = .5).

### Procedure

This study employed a cross-sectional design to examine differences between students with and without SLD in academic, emotional, social, and executive functioning challenges. Participants were recruited by posting an advertisement on Facebook, specifically within groups targeted at students with and without SLD. In addition, participants were recruited through personal networks by distributing the advertisement via WhatsApp. After providing informed consent, participants completed an online survey assessing demographic information, as well as self-report questionnaires. Additionally, they completed a writing task as depicted below. It took about 30 minutes to complete the survey. Compensation was given as course credit or monetary payment. Data were collected anonymously to ensure confidentiality and analyzed to compare students with and without SLD across the variables of interest. The minimal anonymized dataset necessary to replicate the study findings is available in the Open Science Framework (OSF) repository: https://doi.org/10.17605/OSF.IO/V9JS4.

### Measures

#### Questionnaires.

Demographic Questionnaire. Participants completed a demographic questionnaire that collected basic background information, including age, gender, and socioeconomic status. In addition, participants were asked about their learning status by responding to the question: “Have you been diagnosed with a specific learning disability (SLD) or Attention Deficit Hyperactivity Disorder (ADHD)?” Participants could indicate whether they had a formal diagnosis of SLD, ADHD, both, or neither. This information was used to classify participants into groups. The demographic characteristics of the sample are presented in Table 1.

**Table 1:** Demographic characteristics of the overall sample (*N* = 95).

		n	%
Gender	Male	25	26%
	Female	70	74%
Education	BA	86	91%
	MA	8	8%
	Ph.D.	1	1%
Language	Arabic	6	6%
	Hebrew	88	93%
	Other	1	1%
LD and/or ADHD	Yes	46	48%
	No	49	52%
SES	Below average	63	66%
	Average	21	22%
	Above average	11	12%

***The Depression Anxiety Stress Scale*** *[DASS;* 51*]* assesses symptoms of depression, anxiety, and stress. The depression scale includes items like “I felt I had lost interest in just about everything,” while the anxiety scale focuses on physical and emotional reactions such as “I had difficulty breathing.” The stress scale measures responses like nervous tension and irritability (e.g., “I found it difficult to relax”). Respondents rate each item on a 0 (did not apply to me at all) to 3 (applied to me very much or most of the time) scale based on their experiences in the past week. Cronbach’s α in this sample was.81 for depression,.83 for anxiety, and.91 for stress.

***The Perceived Stress Survey*** *[PSS;* 52*]* includes 14 items assessing how stressful participants perceive their lives (e.g., “In the last year, how often have you felt nervous and stressed?” “How often have you been upset by something unexpected?”). Responses are rated on a 5-point Likert scale from 0 = never to 4 = very often. In this study, Cronbach’s α for the PSS was.78.

***The Ruminative Responses Scale*** *[RRS;* 36*]* includes 22 items assessing three aspects of rumination: depression-related thoughts, brooding, and reflective pondering (e.g., “I think about how sad I am” and “I think about how alone I feel”). Responses are rated on a 4-point scale from 1 (almost never) to 4 (almost always). In this study, the scale showed high reliability with a Cronbach’s α of.94.

***Writing Task***. Participants completed a writing task adapted from previous studies [53,54]. They were instructed to recall and write about a recent negative academic event, and to answer self-reported questions of their current emotional state. Specifically, participants were asked to describe a distressing academic experience from the past weeks using this prompt: “Recall a recent college event (within the last 2-4 weeks) that made you feel regretful, depressed, or bad about yourself. Describe what happened, and how you felt and thought afterward.”

### Analytical strategy

Two members of the research team coded participants’ emotional events based on May and Stone’s [55] method. Events were categorized into four groups based on common SLD-related difficulties in higher education (Emotional, social, academic and EF). A third judge resolved discrepancies. Frequency data for each category were entered into SPSS, where chi-square tests assessed differences between the groups in the number of reported challenges. Independent t-tests were used to compare rumination between SLD and non-SLD students. Only factors with significant group differences were analyzed as moderators, and moderation analysis was performed to examine the impact of these challenges on the relationship between SLD and rumination.

## Results

### Differences in rumination and emotional distress (PSS and DASS) between students with and without SLD

In order to examine differences between groups in terms of emotional distress and their tendency to use rumination, independent t-tests were applied. The results revealed that students with SLD reported significantly higher levels of rumination (*M* = 55.04, *SD* = 15.26) compared to students without SLD (*M* = 48.53, *SD* = 14.73), *t*(93) = −2.12, *p* < .05, Cohen’s *d *= .43. Also students with SLD reported significantly higher perceived stress (PSS) levels (*M* = 38.74, *SD* = 5.38) compared to students without SLD (*M* = 35.47, *SD* = 5.75), *t*(93) = −2.86, *p* < .05, Cohen’s *d *= .59. However, no significant differences were found in the DASS, *t*(93) = −1.73, *p* = .086.

### Group differences in higher education challenges

Following the event coding, a chi-square test was applied in order to examine differences in academic, emotional, social, and EF challenges between students with and without SLD. Students with SLD reported more EF difficulties in comparison to their peers without SLD, χ²(1) = 4.58, *p* < .05. However, no significant differences were found between groups in the academic, emotional, and social challenges (*p* = 0.4; *p* = .57; *p* = 1, respectively).

### The mediating role of EF in the link between SLD and rumination

Following the results of the chi-square test showing that only the EF challenges variable was significantly different between groups, a moderation analysis using the PROCESS macro [56, model 1] examined whether EF moderated the link between SLD and rumination. In this model, SLD was the independent variable, rumination the dependent variable, and EF the moderator. The overall model explained 9.78% of the variance in rumination, *F*(3, 91) = 3.29, *p* = .024, *R²* = .098, indicating that the combination of SLD, executive functions, and their interaction significantly predicts rumination. The direct effect of SLD alone on rumination was non-significant, *b* = 1.32, *SE* = 3.84, *t*(91) =.34, *p* = .732, 95% *CI* [−6.30, 8.94]. Similarly, the effect of EF on rumination was also non-significant, *b* = −3.13, *SE* = 4.77, *t*(91) = −.66, *p* = .513, 95% *CI* [−12.60, 6.34]. However, the interaction between SLD and EF approached significance, *b* = 12.69, *SE* = 6.46, *t*(91) = 1.96, *p* = .053, 95% *CI* [−.15, 25.53], indicating a possible moderating effect of EF on the link between SLD and rumination.

To further understand the interaction, conditional effects of SLD on rumination were examined at different levels of EF. When EF difficulties were absent, SLD was not a significant predictor of rumination, *b* = 1.32, *SE* = 3.84, *t*(91) =.34, *p* = .732, 95% *CI* [−6.30, 8.94]. However, when EF difficulties were present, SLD significantly predicted higher levels of rumination, *b* = 14.01, *SE* = 5.20, *t*(91) = 2.69, *p* = .008, 95% *CI* [3.68, 24.34].

## Discussion

Recently, the enrollment of students with SLD in higher education has steadily increased [3,5,6]. This study examined differences between students with and without SLD in academic, emotional, social, and executive function (EF) challenges, as well as in levels of rumination and emotional distress. It also explored how these challenges are associated with rumination. Importantly, this study is the first to assess whether EF challenges moderate the relationship between SLD status and rumination. The findings showed that students with SLD reported significantly greater EF difficulties, higher levels of rumination, and increased perceived stress compared to their non-SLD counterparts. However, no significant group differences were observed in academic, emotional, social difficulties, or distress (psychological distress and perceived stress). Notably, EF challenges moderated the association between group and rumination, such that students with SLD who experienced EF challenges reported particularly high levels of rumination.

These findings align with prior research linking SLD with EF challenges. Research has consistently shown that students with SLD may struggle with initiating tasks, organizing information, shifting between tasks, and maintaining sustained attention [9,16,57]. These EF difficulties are not merely secondary to academic challenges but are often intrinsic to the neurological and cognitive profiles of individuals with SLD. For example, weaknesses in working memory can impair the ability to follow multi-step instructions or retain information presented in lectures [58], while deficits in inhibitory control may result in distractibility or difficulty filtering out irrelevant stimuli [59]. In the context of higher education, where academic success depends heavily on independent learning, time management, and adaptive problem-solving, these EF impairments can create significant barriers.

The absence of significant group differences in academic, emotional, or social difficulties diverges from earlier studies [e.g., 6,8], which reported greater struggles among students with SLD. One possible explanation is that students with SLD in the current sample may have developed effective compensatory strategies or institutional support, such as using assistive technologies, or received substantial institutional accommodations. Alternatively, self-selection bias could have played a role, with more resilient and higher-functioning students choosing to pursue higher education and to participate in the study. Future research should further investigate the role of support services and individual coping resources in shaping the experiences of students with SLD.

Consistent with expectations, students with SLD reported higher levels of both rumination and perceived stress. This finding aligns with previous work showing elevated rumination among children with SLD [19,60]. These students often face persistent difficulties in managing academic demands and cognitive demands such as organizing tasks, maintaining attention, and processing information efficiently [14,16]. Such challenges may contribute to a heightened sense of frustration and failure, fostering negative thinking patterns and increasing stress levels. Previous research also highlighted elevated stress levels and reduced school engagement among students with SLD, impacting their mental health and academic performance [61,62]. The daily struggle to meet academic expectations in an environment not fully tailored to their learning needs may increase their perception of stress and reduce their sense of competence and control. These cognitive and emotional challenges can further lead to greater emotional distress and reinforce negative emotional patterns, such as persistent rumination, which has been shown to intensify negative affect and impair problem-solving [6,29].

A key contribution of this study is identifying EF difficulties as a moderator in the relationship between SLD and rumination. While SLD alone did not predict rumination, students with both SLD and EF challenges reported significantly higher rumination levels. This supports cognitive models of rumination suggesting that deficits in inhibitory control and cognitive flexibility impair individuals’ ability to disengage from negative information [3,42,63]. The impaired disengagement hypothesis [42] offers a compelling framework: EF deficits likely trap students with SLD in cycles of negative thinking by making it harder to shift attention away from failure-related thoughts. Furthermore, the high comorbidity of ADHD symptoms among participants may have amplified EF difficulties [64,65], further increasing vulnerability to rumination.

### Limitations

This study has several limitations. First, self-reported data may introduce bias, such as social desirability or recall issues, though it provides valuable insights into subjective experiences like rumination and social challenges. Second, the predominantly female sample may influence the results. However, this reflects the gender distribution in higher education (Central Bureau of Statistics, Israel, 2021). Third, relying on self-reported SLD without formal diagnoses may affect accuracy, so future studies should use professional evaluations. Lastly, that coding was based on one event only, what may limit sensitivity to group differences. Asking for more events could provide deeper insights. Finally, the statistical power of the study based on the sample size was relatively low (67%), what may have limited the ability to detect meaningful effects. Future research should consider larger sample sizes to enhance the reliability and generalizability of the results.

## Conclusion

This study offers both theoretical and practical insights. Theoretically, it extends the understanding of emotional difficulties among students with SLD by highlighting the possible role of EF vulnerabilities in exacerbating rumination. Rather than viewing SLD as a direct predictor of emotional distress, the findings suggest that cognitive processes, particularly EF deficits, are key mechanisms linking learning difficulties to maladaptive emotional outcomes. Practically, the results emphasize the need for interventions targeting EF skills in students with SLD. Enhancing EF — such as improving planning, organizational skills, and attentional control — may help reduce ruminative thinking and emotional distress. Cognitive training programs [e.g., 66,67] and tailored cognitive-behavioral therapy (CBT) interventions focused on improving cognitive flexibility and emotion regulation could be particularly beneficial. Universities and colleges might also consider integrating EF skill-building workshops into their academic support services.
