# Supplementary material for: Challenges in higher education: Differences between students with and without specific learning disorder and the moderating role of executive functions
Source: PLoS One. 2025 Sep 5;20(9):e0331375. doi: 10.1371/journal.pone.0331375 (PMC12412975; doi:10.1371/journal.pone.0331375)
Supplement: Attachment — Submitted filename: letter to the reviewer 22.6.25.docx [file pone.0331375.s001.docx]

Dear Dr. Srikanth Goud,

We are happy to submit a revision of our paper titled Challenges in Higher Education: Differences between Students with and without Specific Learning Disorder and the Moderating Role of Executive Functions (PONE-D-25-07350). We thank you and the reviewers for your thoughtful and illuminating notes. We have carefully addressed all of the reviewers' concerns, particularly regarding the introduction and the discussion. All edits have been made using “Track Changes,” and any new additions to the article are additionally highlighted in turquoise for clarity.

We believe these revisions have significantly strengthened the manuscript.

Thank you for considering our manuscript for publication in *PLOS ONE*. We are looking forward to hearing from you.

Sincerely,

**Marlyn Khouri**

Department of Special Education, Faculty of Education, University of Haifa, Israel

+972508851741

[Mkhouri89@gmail.com](mailto:Mkhouri89@gmail.com)

**Atheer Massarwe**

Department of Special Education, Faculty of Education, University of Haifa, Israel

+972504839393

m.atheer1996@gmail.com

**Noga Cohen**

Department of Special Education, Faculty of Education and the Edmond J. Safra Brain Research Center for the Study of Learning Disabilities, University of Haifa, Israel

+972545852486

noga.cohen@edu.haifa.ac.il

**Journal requirements:**

1. Please ensure that your manuscript meets PLOS ONE's style requirements, including those for file naming. The PLOS ONE style templates can be found at:

https://journals.plos.org/plosone/s/file?id=wjVg/PLOSOne_formatting_sample_main_body.pdf and <https://journals.plos.org/plosone/s/file?id=ba62/PLOSOne_formatting_sample_title_authors_affiliations.pdf>

Response: Thank you for the reminder. We have reviewed the journal’s formatting and style instructions, including file naming requirements, and have revised the manuscript accordingly to ensure full compliance with PLOS ONE’s guidelines.

2. Please note that PLOS ONE has specific guidelines on code sharing for submissions in which author-generated code underpins the findings in the manuscript. In these cases, we expect all author-generated code to be made available without restrictions upon publication of the work. Please review our guidelines at <https://journals.plos.org/plosone/s/materials-and-software-sharing#loc-sharing-code> and ensure that your code is shared in a way that follows best practice and facilitates reproducibility and reuse.

Response: All data and analysis code (SPSS file) underlying the findings of this study are available on the Open Science Framework (OSF) at: [https://osf.io/v9js4/].

3. Please provide a complete Data Availability Statement in the submission form, ensuring you include all necessary access information or a reason for why you are unable to make your data freely accessible. If your research concerns only data provided within your submission, please write "All data are in the manuscript and/or supporting information files" as your Data Availability Statement.

Response: Thank you for the reminder. We have updated the submission form to include a complete Data Availability Statement. As our data are publicly available via the Open Science Framework (OSF), we included the following statement: 'All data are available on the Open Science Framework (OSF) at [https://osf.io/v9js4/*]*.

4. PLOS requires an ORCID iD for the corresponding author in Editorial Manager on papers submitted after December 6th, 2016. Please ensure that you have an ORCID iD and that it is validated in Editorial Manager. To do this, go to ‘Update my Information’ (in the upper left-hand corner of the main menu), and click on the Fetch/Validate link next to the ORCID field. This will take you to the ORCID site and allow you to create a new iD or authenticate a pre-existing iD in Editorial Manager.

Response: Thank you for the reminder. I have updated my information in the PLOS ONE Editorial Manager system and successfully added and validated my ORCID ID.

5. Please amend your authorship list in your manuscript file to include author Marlyn Khouri, Noga Cohen, Atheer Massarwe.

Response: Thank you for your comment. The manuscript file has been updated to include the full authorship list, now reflecting Marlyn Khouri, Noga Cohen, and Atheer Massarwe.

6. Please ensure that you refer to Figure 1 in your text as, if accepted, production will need this reference to link the reader to the figure.

Response: Thank you for pointing this out. The reference to Figure 1 remained in the manuscript by mistake after we decided not to include the figure. We have now removed the reference from the text to avoid confusion.

7. Please include a copy of Table 1 which you refer to in your text on page 5.

Response: Thank you for your comment. Table 1 is included in the manuscript file and has also been uploaded as a separate file in the submission system, as required.

**Reviewer's Comments**

1. Introduction needs more details. The sentence flows are not in order that connect the story well.

Response: We have revised the introduction and added further details to improve its clarity and flow. We hope it now presents the content more coherently.

2. "Fostering a sense of inadequacy and hopelessness", is it right to use foster here?

Response: As part of the revisions made to the introduction, the word 'fostering' was removed.

3. I could not understand the main objective of this study. Yes. There will be challenges with and without SLD. Period. But what next? What was the main goal to carry out this study?

Response: As mentioned in the paragraph preceding the Methods section, this study compared academic, social, emotional, and executive function (EF) challenges between students with and without SLD and examined which of these challenges might moderate the relationship between SLD and rumination. These objectives form the central aims of the study. Previous research has shown that students with SLD tend to experience greater difficulties in academic, emotional, social, and EF domains. Studies have also found that students with SLD report higher levels of rumination compared to their peers; however, the reasons for this heightened rumination remain unclear. Therefore, the main goal of the current study was to identify which of these specific challenges may explain or moderate the association between SLD and increased rumination. We have made this clearer in the text.

4. This sentence is not clear – “Hence, the study examines which of the difficulties might moderate the link between SLD and rumination.”

Response: This sentence was originally intended to explain the aim of the study; however, it was removed during the revision of the introduction.

5. Paper needs a separate literature review section

Thank you for your comment. In preparing the manuscript, we closely followed the formatting and structural guidelines provided by PLOS ONE, which do not require a separate literature review section.

6.  Introduction section must be written like an introduction section. Too many references make the reading difficult.

As noted above, we revised the Introduction to improve clarity. We also adjusted the in-text citations and reference list to align with the journal’s formatting guidelines (using numbered citations), and removed references that were deemed non-essential.

7. 67% power, which isn't great, especially in research where we want to be confident in our findings. Justify.

Response: While the statistical power of this study was 67%, which is below the conventional 80% threshold, it is important to consider the constraints and goals of the research. This study focused on a specific and often underrepresented population—students with SLD—which inherently limits sample size due to recruitment challenges. Despite the reduced power, the study still detected significant effects, suggesting that the observed relationships are meaningful and not purely due to chance. Additionally, the findings are consistent with existing theoretical frameworks and prior research, as presented in the manuscript, further supporting their validity. Future studies with larger samples are encouraged to replicate and extend these findings. However, we refer to this issue in the limitations section.

8. Research design section is not clean. Explain sampling method. Give a proper context for the study. What is the study design? Explain the methodology and methods.

Response: the method section has been revised to provide clearer information regarding the sampling method, study design, and overall methodology. We hope these revisions enhance the clarity and coherence of the study.

9. More details are needed for certain test results. Example for cronbach alpha. Put them in order.

Response: Thank you for your comment. We have reported the Cronbach’s alpha values for the questionnaires used. However, we were unsure if the reviewer is requesting additional details beyond those already included. If there are specific results or statistics the reviewer would like us to expand upon or reorder for clarity, we would be happy to make those revisions.

10. Why 4 point scale? Why not 5? Any specific reason?

Response: Thank you for your question. Could you please clarify which specific scale you are referring to?

Regarding the Ruminative Response Scale (RRS), we used the original 4-point Likert scale format developed by Nolen-Hoeksema & Morrow (1991). This 4-point scale was intentionally designed without a neutral midpoint to reduce socially desirable responding and to encourage participants to reflect on their actual behavior rather than defaulting to a neutral option. Therefore, we adhered to the validated 4-point format to maintain consistency with the scale’s original psychometric properties and previous literature using this instrument.

Similarly, the Depression Anxiety Stress Scales (DASS; Lovibond & Lovibond, 1995) also uses a 4-point Likert scale, and we followed the original format as recommended by the authors.

In contrast, the Perceived Stress Scale (PSS; Cohen et al., 1983) uses a 5-point Likert scale. We adhered to this original 5-point format as it is standard for this instrument and supported by the literature.

Overall, we used the validated and widely accepted scale formats for each measure to ensure reliability, comparability, and fidelity to the original instruments.

11. Results need to be presented well. Explain with flow on why each of the tests was done. Please keep the methods section clean. Most data is not presented in the study and needs more graphs. Use more tables and plots.

Response: We believe that the statistical values are important for replicability purposes. Whenever possible, we moved the values to tables and removed them from the text.

12. Discussion section is weak. The findings are not inferred well. I wonder if the discussion is work discussion or a literature survey.

Response: Thank you for this important comment. The discussion section has been revised to enhance clarity and coherence. We have ensured that all findings are explicitly addressed and interpreted in light of relevant literature. The revised version aims to provide a balanced integration of empirical results and theoretical context, in line with standard expectations for a discussion section.

13.  A lot of things in discussion do not connect well with the study and the study objective.

Response: Thank you for your feedback. As mentioned above, the discussion section has been revised for greater clarity and focus. We made specific efforts to ensure that all interpretations are closely aligned with the study’s objectives and findings, and that the discussion remains relevant and well-integrated throughout.

14. Why put limitations in discussion?

Response: Thank you for your feedback. The limitations are already presented in a dedicated paragraph within the discussion section. To enhance clarity and structure, we have now added a subheading titled **“Limitations”** to clearly signal this part of the discussion.

15. Conclusion must be separate section

Response: Thank you for the suggestion. As the journal allows for a separate Conclusions section, we have revised the manuscript accordingly and now present the conclusion as a distinct section, in line with the reviewer’s recommendation.
